# Development of a market-driven training model for rural women in Iran by using a qualitative paradigm

**DOI:** 10.3389/fsoc.2024.1339101

**Published:** 2024-05-14

**Authors:** Zeynab Allahmoradi, Seyed Jamal Farajallah Hosseini, Farhad Lashgarara, Reza Moghaddasi

**Affiliations:** Department of Agricultural Economics, Extension and Education, Science and Research Branch, Islamic Azad University, Tehran, Iran

**Keywords:** market-driven training, Kurdistan Province, rural women, agricultural extension, grounded theory

## Abstract

**Problem:**

Training programs aimed at empowering rural women in Kurdistan, Iran, have fallen short of their goals. This study offers a unique understanding of how contextual factors and cultural nuances impact the effectiveness of market-driven training programs for rural women in Kurdistan.

**Objectives:**

This study explored factors affecting the effectiveness of market-driven training programs for rural women in Kurdistan.

**Methods:**

A qualitative approach using grounded theory methodology was employed. Interviews were conducted with 23 key informants, including rural women and experts. Data analysis with MAXQDA software identified seven categories and a core category related to market-driven training.

**Findings:**

The study revealed that successful implementation of market-driven training hinges on various external and internal factors. These include access to markets, facilities, and funding, along with effective monitoring of business activities. Additionally, rural community culture and the purchasing power of women were identified as key intervening conditions impacting program success. When implemented effectively, market-driven training programs have the potential to empower women, reduce rural migration, and improve product quality.

**Recommendations:**

The research suggests that future training programs should adopt a multi-dimensional approach that addresses the identified factors to achieve sustainable positive outcomes for rural women in Kurdistan.

## 1 Introduction

Globally, empowering rural women is crucial for agricultural development and poverty reduction. They represent a significant portion of the agricultural workforce yet face numerous challenges hindering their economic participation. These include limited access to resources, education, and market opportunities. In developing countries like Iran, these challenges are further compounded by cultural norms and social inequalities. Kurdistan province, with its low human development index and high rural female unemployment rate, exemplifies this struggle. Existing training programs aimed at empowering rural women have fallen short, highlighting the need for a more effective approach.

Despite a decline in overall farmer numbers, the number of women farmers is on the rise. This trend coincides with a growing interest in organic practices and sustainable agriculture, areas where women are increasingly visible (Sudimack, 2021). However, this rise does not come without challenges. Rural women often juggle household responsibilities with discrimination and a lack of economic empowerment, especially in developing countries (Elsayed and Roushdy, 2017; Kapur, 2019). Their dependence on husbands’ income further restricts their independence (Aghdasi et al., 2023). Despite limited research on women in agriculture across different cultures (Surangi, 2022), it’s clear that supporting these women is crucial for their economic growth and overall well-being.

Traditionally, women in low to middle-income countries account for half of the agricultural labor force and suffer long-term considerable gender bias in rural societies (Akter et al., 2017; Sell and Minot, 2018). Women have demonstrated a unique ability to connect farms and ranches with social resources, drive change and adaptation in agriculture, facilitate farm and ranch succession, and build community after a disaster. The capacity of agriculture and rural communities to adapt in the face of significant adversity depends on those unique abilities (Bertsch, 2020).

In terms of quantities of assets, agricultural inputs, and resources, men have more control over their resources compared with women (Seuneke and Bock, 2015; Doss et al., 2018). Additionally, women farmers have less access to land, information, capital and credit, and other inputs (FAO, 2011; Colfer et al., 2015). However, there is an argument that giving rural women more opportunities to control their income, would eventually contribute to the livelihood of rural families. However, their participation in income generating activities (IGAs) is a crucial mechanism for ensuring the rural development of developing countries (Alemu et al., 2022). It would be achieved through market-oriented training and governmental strategy considering putting necessary resources into rural area (Kabir et al., 2019). But studies show that there is a considerable challenge to overcome the existing barriers for rural women (Doss et al., 2018).

Researchers have pointed out that there is significant inequality between men and women in controlling household income in rural areas (Colfer et al., 2015; Akter et al., 2017; Doss et al., 2018). It is important to point out that women are the ones primarily responsible for the work within the home and farm in the less developed regions of the world (Seuneke and Bock, 2015; Doss et al., 2018). It is well-documented that one of the main pillars in the empowerment of rural women is to create an environment so they could achieve their financial independence (Colfer et al., 2015; Akter et al., 2017; Doss et al., 2018; Gupta et al., 2019).

One of the concerns of the international community is the inequality between men and women in the field of education (McLain, 2018). This trend has contributed to the declining quality of life among rural population. The type of training for rural women should be based on their demands and needs. In many developing countries, there has been a lot of emphasis on changing educational systems based on learner demand (Ramasamy and Pilz, 2020).

Therefore, there is a need for training rural women about financial issues, but in terms of market-oriented training programs, they are being neglected as a recipient of these training programs (Shan et al., 2015). This would reduce their bargaining power in selling the products and buying inputs (Coles and Mitchell, 2010).

A well-organized advisory service should organize in a manner that offers appropriate market-oriented training to rural women (IFAD, 2010; FAO, 2011; Cassar and Katz, 2016). The term market orientation (MO) refers to which an organization applies marketing concepts in the process of providing its services (Hoa et al., 2018). It especially requires specific actions that respond to consumers’ needs (Khatiwada et al., 2017). However, the multiple roles that rural women should carry in their households require a sustainable, flexible, and accessible market-oriented training program (FAO, 2011; Seuneke and Bock, 2015; Shan et al., 2015). Market-Driven Training programs for rural women should provide the consumer with greater satisfaction from products and help the firm to achieve performance goals.

According to Bhattarii et al. (2019) market orientation and market, disruptive capability are the two marketing-related concepts within the existing literature. Market orientation as a mechanism is used to provide information on consumers and competitors in the target markets (Kumar et al., 2011; Line and Wang, 2017; Rezaei et al., 2017; Byrne et al., 2018; Gupta et al., 2019).

A growing body of evidence suggests that market-oriented training program has consistently been demonstrated to positive effect on several important development programs under different conditions (Devaux et al., 2009; Hoa et al., 2018).

The results of a study about the role of market-oriented agri-technology and agri-extension services in Malaysia show that those farmers who underwent the training classes have attained the knowledge and skills necessary to adopt the technologies and be more confident in running their businesses (Disosdado et al., 2019). The market-oriented training program would facilitate the access of rural women to markets and enhance their livelihoods (Combaz, 2013; Seuneke and Bock, 2015; Shan et al., 2015; Khatiwada et al., 2017; FAO, 2019). Despite the potential capacities of the market-oriented training program in the rural area, it has failed to utilize this potential to empower rural women (Combaz, 2013; Seuneke and Bock, 2015).

However, a vast number of studies about the class and ethnic identities of the rural population have neglected the needs of rural women (Ghouse et al., 2017; Raghuvanshi et al., 2017). Rural women in Iran like many developing countries, due to gender discrimination, have not been able to enhance their technical knowledge and management skills (Ghouse et al., 2017; Savari et al., 2020).

Women constitute the largest proportion of the rural adult South African population. The rural areas host women with the lowest levels of education and skills training in the country. The lack of basic education and skills has resulted in many of them being marginalized, rejected and discriminated against, unemployed and living in poverty. These social problems have serious negative effects on them and their families, which is why the educational provision of Adult and Community Education, and Training in South Africa is a significant tool for their socio-economic advancement (Caagbay, 2019).

According to available data, there are ten million rural and nomadic women in Iran. Based on Agricultural the last statistics, six million rural women with an average age of 18 years old, have officially been classified as active workforce in Iran. Additionally, based on the latest Statistics, only 229,153 rural women work in the agricultural sector.

Kurdistan Province is one of the least developed areas in Iran, which is ranked 21st among 31 provinces (Statistics Center of Iran, 2016).

Although training programs were implemented in rural areas of Kurdistan, the assessment of these training programs shows their inadequacy and ineffectiveness in reaching the objectives.

In this study, the perspectives of experts about internal and external factors that affect the implementation of market-driving training programs were explored and a model was designed based on the results of the grounded theory analysis. According to the FAO Women’s Section in the United Nations, a quarter of the world’s population and half of the active population in rural communities are rural women. They are the most invisible participants in the economic process of society and family. According to the results of the 1,395 census, the population of women in Iran is about 3,400 thousand people. The share of rural women in the population is 10,200,00 of the total population. Approximately 470,000 people, or about 29% of the population of about 1,600,000 people in Kurdistan Province, live in rural areas. The population of rural women in the province is about 230,000 and of rural men is about 240,000 (Statistics Center of Iran, 2016).

Kurdistan province consists of 10 cities, 31 districts, 29 counties, and 86 rural districts and has a 230 km land border with Iraq. The rate of urbanization in the province has increased from about 40% in 1,365 to 71% in 95. The growth of urbanization is a function of factors, the most important is the conversion of villages into cities and the migration of villagers to urban areas due to unbalanced urban–rural development.

Kermanshah, Ilam, Chaharmahal, Bakhtiari, Kurdistan, Ardabil, and Gilan provinces have the lowest employment rates for young women (less than 50%). The lowest literacy rates for women belong to the provinces of Sistan and Baluchistan (71% illiterate) and Kurdistan (70% illiterate). The economic participation rate of rural women over the age of 10 is 10.8%. The unemployment rate of rural women in Kurdistan province is reported to be 3.13%, which is higher than the national average and is among the top 10 provinces in the country. The province’s human development index is one of the most underdeveloped provinces in the country. This province is ranked 29th in terms of the human development index. Youth unemployment is over 25%. Kurdistan is the fifth province in the country in terms of the number of unemployed populations, while this province has 2% of the country’s population and less than 1% in GDP and income. It’s *per capita* is less than half of the national average *per capita* income.

The income crisis in Kurdistan has disproportionately impacted rural communities, particularly women. This economic hardship disrupts their livelihoods and exposes them to the brunt of environmental changes. Furthermore, gender inequalities exacerbate their vulnerability, making them more susceptible to mental health issues like depression and anxiety (Cobano-Delgado and Llorent-Bedmar, 2020). In essence, Kurdish rural women face a multitude of challenges that threaten their overall well-being.

Challenges such as a low human development index and consequently increased migration, turning to fake jobs, and potentials such as a significant population of rural women active in agriculture, livestock, handicrafts, access to border markets, and Indigenous and cultural attractions which require responsible and forward-looking planning. Economic empowerment of women creates positive effects on self-confidence, and bargaining power, promoting their position within the family and society and increasing the effective presence of women in the decision-making process. Improving the conditions of education and employment of women is the key to the success of measures to improve rural and nomadic development. So far, market-driven extension and training programs have not been considered a priority, and issues such as economic fluctuations and changing customer preferences require more responsiveness to the market-oriented training program. The rural women’s education program should be considered a driving force to achieve financial empowerment for rural women.

The main question in this study is to find out factors influence the effectiveness of market-driven training programs for rural women in Kurdistan province, Iran. The objectives of the study are to explore the factors affecting the effectiveness of market-driven training programs for rural women in Kurdistan province, Iran; and to develop a market-driven training model tailored to the needs of rural women in Kurdistan province, Iran.

## 2 Methodology

### 2.1 Research design

The current study was conducted using a qualitative approach to investigate and describe market-oriented training and provide a theoretical model. The reason for using the qualitative research method is that the issue of sustainability is not a one-dimensional issue that can only be investigated with a quantitative method. Due to the social, cultural, and epistemological aspects governing this issue, it is necessary to conduct a qualitative study and identify the thoughts, descriptions, statements and opinions of experts. Qualitative research can be used in many different settings and encompasses multiple methodologies based on phenomena and issues with limited literature. Qualitative research helps the researcher provide an in-depth understanding of the phenomena and at the same time it gives a rich picture of non-quantitative information about the issue (Polit and Beck, 2010).

The grounded theory methodology (GT) was particularly well-suited for this study due to the limited research on market-driven training (MDT) for rural women. This approach allowed the model to emerge from the data itself. The research employed MAXQDA software to facilitate data analysis, which involved: thorough review of the data to identify recurring themes; coding these themes using keywords and phrases; grouping the codes into hierarchical concepts; and identifying relationships between the concepts to develop a comprehensive model.

This study employed a grounded theory (GT) approach to analyze data collected through individual interviews. GT is an inductive research method that allows theories to emerge from the data itself, rather than applying pre-existing frameworks (Bytheway, 2018; Milani and Hashemi, 2020). This research design is particularly well-suited for situations, like this study, where there is limited existing research on the topic.

Through theoretical saturation, we arrive at a point where no further data reveals substantially new insights that would change our evolving theory. This signifies that we have grasped the central concepts underlying the phenomenon we are investigating.

Thus, this study adopted a grounded theory (GT) research design to analyze the data collected through individual interviews. The grounded theory procedure by Corbin and Strauss (2008) was used to investigate the perception of respondents about the MDT for rural women. This method is used to describe the structure and process of phenomena. This process finally leads the researcher to develop a data-based model comprising six categories (Soltani et al., 2020).

### 2.2 Participants and sampling strategy

The purpose of qualitative research is to gain a deeper understanding of a phenomenon, rather than to generalizing the findings. Therefore, careful selection of research samples can help us conduct a more thorough evaluation (Naderifar et al., 2017).

Snowball sampling is arguably the most widely employed method of sampling in qualitative research in various disciplines across the social sciences. It is sometimes used as the main vehicle through which informants are accessed, or as an auxiliary mean, which assists researchers in enriching sampling clusters, and accessing new participants and social groups when other contact avenues have dried up (Noy, 2008).

This qualitative research employed purposive sampling to identify a group of experts on rural women and market-driven training programs. The sample size reached theoretical saturation at 23 participants. For the interview process, we selected participants who met the following criteria: a minimum of 10 years of experience working with rural women, being at least 40 years old, and possessing a master’s degree in a relevant field.

This study used purposive and snowball sampling techniques to select the sample, while it relies on referrals from initial subjects to generate additional subjects. Such sampling continued until the saturation of the data. The data from the interview reached saturation through in-depth individual interviews with 23 participants. A minimum of five participants is necessary to adequately reflect the nature of a given experience (Hussein et al., 2020).

Table 1 summarizes a demographic description of respondents. Among 23 respondents, 5 of them were female and 18 were male. It was reported that 21 participants were married, and their ages ranged from 40 to 60 years. The majority had relatively high-level of educational backgrounds. The respondents had experiences in market-oriented training and rural women empowerment from Tehran University, Kurdistan University, the Director General of Rural Women Affairs of the Ministry of Jihad Agriculture, the Director General of Rural Cooperatives of Rural Women, Private Sector Managers Acting in the Field of Education and Employment of Rural Women and Two Successful Rural Women in Kurdistan province.

**Table 1:** Profile of respondents (*n* = 23).

No	Sex	Marital status	Age	Education	Work experience
1	M	Married	51–60	Ph.D. Degree	Over24
2	M	Married	41–50	Ph.D. Degree	15–19
3	F	Married	41–50	MSc Degree	20–24
4	M	Married	Under40	MSc Degree	Under14
5	M	Single	Under40	MSc Degree	Under14
6	M	Married	51–60	Ph.D. Degree	Over24
7	M	Married	Over60	Ph.D. Degree	Over24
8	M	Married	41–50	Ph.D. Degree	20–24
9	M	Married	51–60	Ph.D. Degree	Over24
10	M	Married	51–60	Ph.D. Degree	20–24
11	F	Single	Under 40	MSc Degree	15–19
12	M	Married	41–50	MSc Degree	15–19
13	M	Married	41–50	Ph.D. Degree	15–19
14	M	Married	51–60	Ph.D. Degree	Over24
15	M	Married	41–50	Ph.D. Degree	15–19
16	M	Married	41–50	Ph.D. Degree	15–19
17	M	Married	41–50	Ph.D. Degree	15–19
18	M	Married	51–60	Ph.D. Degree	Over24
19	M	Married	51–60	Ph.D. Degree	Over24
20	M	Married	Over60	Ph.D. Degree	Over24
21	F	Married	41–50	MSc Degree	20–24
22	F	Married	51–60	Ph.D. Degree	Over24
23	F	Married	41–50	MSc Degree	20–24

### 2.3 Interview strategy

Data were gathered through in-depth interviews, observations, and group discussions. Unstructured interviews were performed with follow-up questions with group discussion until “reaching saturation.” Each interview lasted about 50 to 60 min. In total, it took 27 days to conduct all the interviews. Interviews were conducted in private settings at times convenient for each participant.

### 2.4 Data coding and analysis

Data analysis was based on Corbin and Strauss (2008) method. It relies on open, axial, and selective procedures. To enhance the trustworthiness of the research findings, we employed memoing and focused coding throughout the data analysis process. Additionally, we ensured the study’s relevance by carefully selecting participants with experience and expertise directly related to the research topic.

Through open coding, the transcribed interviews were studied, and data were broken down into discrete parts. Through microanalysis at the open coding stage, each interview was carefully studied line by line, and many concepts related to the main research question were explored, examined, and compared for similarities and differences. In the next stage, namely axial coding, themes with relevant meaning were examined as specific categories. In the next phase, the main categories and sub-categories were linked during the axial coding process. In the third stage, in the selective coding phase, the core category was extracted. Research validity and reliability were confirmed by simultaneously using multiple methods, creating a database, and using member checking when completing and revising the interview texts (Table 2). To analyze the data, the MAXQDA software was used.

**Table 2:** Examples of concepts, subcategories, and extracted categories in the coding stage.

Interview text	Concept	Frequency	Categories
When it comes to market-driven training, the first thing that comes to mind is employment.	Employment	10	Creating a business
Training programs for rural women should be applicable.	Practical	14	Need assessment
Rural women should be trained based on market needs.	Market needs	12	
Before starting the business, the need preferences should be identified.	Market preferences	11	Earn profit
Training should result in the production	Increases production	15	
Training should contribute to increasing income	Increasing income	17	
Training should improve rural family livelihood.	Improving livelihood	13	

## 3 Results

**Figure 1:**
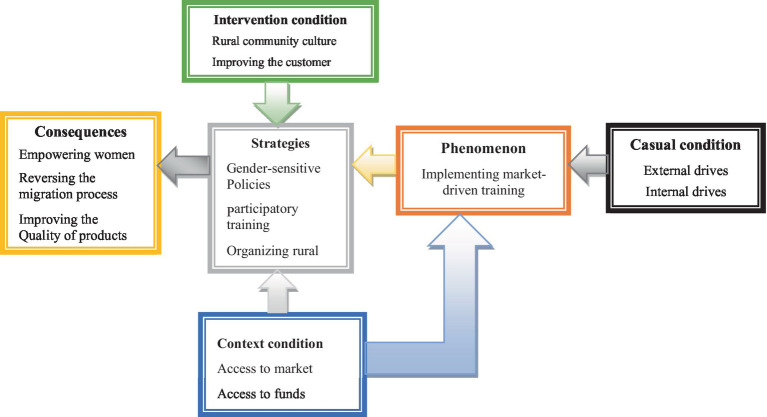
Conceptual Framework.

Table 2 shows the concepts that were extracted from interviews. After extracting the concepts, these units were given a code to reflect those concepts. In the next step, similar concepts were classified into several categories. A total of 350 revealed concepts, 60 subcategories, and 24 categories were extracted. After the identification of categories, selective coding was performed in which the categories identified in the previous stage were integrated and combined to provide a conceptual framework (Figure 1; see also Table 3).

**Table 3:** Some concepts, subcategories, and external factors are derived in the coding stages.

Row	Number of concepts	Sub-categories	Categories	External factors
1	7	Organizing a community of rural women	Organizing	Micro-strategies
2	12	Engaging the Rural women in training	Active training for rural women	
3	8	Organizing rural women based on skills	Classification based on skill	
4	9	Identifying Macro-bureaucracy about rural women’s businesses		Macro-Strategies
5	4	Identifying challenges in existing macro policies related to rural women	Policies in the field of rural women	
6	7	Buying rural women’s products	Guaranteed purchase	Casual conditions (External drive)
7	10	Guaranteeing prices of products	Contract farming	
8	8	Calculating the cost price		
9	7	Providing better financial facilities	Financial support	
10	5	Providing banking facilities		
11	8	Furnishing long-term training	Continues education	
12	7	Organizing Comprehensive training		
13	5	Reducing the amount of production waste due to lack of refrigeration	Product packaging	Casual conditions (Internal drive)
14	8	Improving processing industries		
15	7	Utilizing modern technologies in production		
16	8	Using the Applied research	Production optimization	
17	6	Saving on raw materials		
18	7	Improving processing industries	Product processing	
19	6	Considering the different types of product	Quality of product	
20	5	Branding the products		
21	8	Considering quality management		
22	4	Dominating the culture of patriarchy	Convincing rural men	Intervening condition (Rural community culture)
23	6	Believing in the ability of rural women to run lucrative businesses	Gender inequality between women and men	
24	6	Improving the level of formal education	Increase the literacy level of spouses	
25	8	Promoting the capacity building Increasing the understanding of the identity of rural women	Rural women identity	
26	10	Improving rural women’s communication with each other	Statues of social capital among rural women	
27	7	Strengthening communication with the urban community		
28	9	Having the trust of the local community		
29	10	Improving communication with extension agents		
30	8	Interacting with experts		
31	10	Having access to the market	Access to the target market	Context condition
32	12	Providing physical facilities	Access to facilities and resources	
33	8	Monitoring the actions of rural women	Monitoring the implementation of rural women’s activities	
34	10	Returning of male household members to the rural areas	Reverse the migration process	Consequences (Increase rural family livelihood)
35	12	Willingness to stay in the rural areas		
36	10	Being able to decide on their own	Empowering rural women	
37	11	Increasing the ability to accept responsibility		
38	14	Enhancing the self-determination		
39	7	Creating more jobs for Women		
40	8	Identifying resources	Increase customer choice	
41	7	Fulfilling the consumer satisfaction	Product competitiveness in quality	
42	9	Producing the quality products		
43	11	Reviewing the production cycle		
Sum	350	60	24	–

### 3.1 Casual condition

The respondents stated causal conditions can be viewed as internal and external factors. Casual condition of IMDT for rural women, not only plays a direct role in accessing domestic and export markets but also increases the importance of non-farm activities in the income-generating portfolio of rural households.

#### 3.1.1 Internal factors

The respondents stated eight items as internal factors for IMDT to rural women in the Kurdistan Province. Rural woman literacy: In a rural area in Kurdistan, the numbers of rural women who can read and write are much less than rural men. There is a great gap in illiteracy between men and women in the rural Kurdistan areas. Household income status: Women in rural areas face constraints such as enough financial resources to engage in economic activities. The rural population cannot produce on the small plot of land that they own and mostly provide labor to others for both farm and nonfarm activities inside and outside their villages.

Access to credits and financial facilities: A key tenet of successful rural business is to ensure rural women have adequate access to credits and financial facilities. The credit programs such as Omid Entrepreneurship Fund can provide financial facilities to rural women to set up small. Rural women (producers) who have access to well-designed credit can afford to invest in more profitable and risky businesses. This creates opportunities for them to have access to markets and adopt.

Access to urban areas: Even though millions of women throughout the world contribute to national agricultural output and food security, numerous studies consistently indicate that they have limited access to urban areas. Trust in government institutions: Trust is important for the success of a wide range of rural small businesses that depend on assistance from the public sector. Women’s ability to undertake business activities is influenced by assistance from public institutions.

#### 3.1.2 External factors

These external factors to MDT have been expressed by the respondents are as following:

Reduce administrative bureaucracy: Although regulation plays a significant role in making the best use of public funds and improving the services, it can also create additional obstacles through strict rules and regulations. Opportunities for national and international markets: There should be more support to develop networks of practitioners and experts that can identify the marketplace at both national and international levels and assist the process of the capacity of the building. Among small-scale rural women producers, for instance, rural women’s cooperatives can enable entrepreneurs to realize economies of scale, invest in technology, and penetrate new markets, while agricultural extension agents can provide appropriate information to identify markets and create synergies among rural women’s businesses.

Holding national training workshops: To market the product or service, it is imperative that marketing and sales efforts specifically reach the segment of the population that will buy these products or services. The training could offer the rural women to make better decisions based on a clear understanding of the characteristics and conditions of the products available. Providing training to all members of households: To ensure that rural women participate in the market-driven training program and to encourage the establishment of gender-sensitive businesses, training should be extended to all members of the household. Market-driven training should also provide a series of measures to increase rural women’s participation in the establishment of small-scale businesses, regarding household and farming activities. Furthermore, rural women typically work longer hours than men, when one considers both paid productive and unpaid reproductive and domestic and care responsibilities, so it is necessary to engage all members of the household in reorienting and delivering the new training curriculum.

### 3.2 Core phenomenon

For all the participants emphasized that most training programs for rural women failed to have a market orientation because they favor more general training over specified training. Seven sub-themes were identified as major MDT to rural women. The perception of respondents about the implementation of training takes a managerial view on market orientation and asks how rural woman training programs can operate to improve market orientation.

The first step in identifying rural women’s target market is to understand what kind of products/services will be offered to the consumers. Training programs can contribute to improving the livelihoods of rural women if they increase their productive capacities, and links them to the markets.

One of the major challenges of MDT was the ineffective linkage of farmers to input and output markets. MDT should enable the diversification of skills and transform a low-productivity economy into a more developed economy, to be more competitive.

On the same note, one of the participants said: ‘Targeting the Kurdish rural women’ market is not simply defining who is the primary customer, but the market should be sufficiently large and reachable for rural women. Once the rural women’s knowledge of product appeals is attained by appropriate analysis, they can determine whether that target market is large enough to sustain their businesses on an ongoing basis.

### 3.3 Areas and contexts for IMDT

The participants raised five following sub-themes as contexts to MDT:

Mentoring trends: All implemented MDTs should be followed by action plans that will regularly be updated considering emerging needs and changing contexts. Successful MDP implementation also requires communication at the regional level, and this is a potential area for regional trainers who have better access to rural women. Mentoring and changing certain training program parameters can have both a direct impact on a rural women’s small-scale business and on offering incentives to women to participate in training programs. The supporting policies, which facilitate self-management of land and productive resources, provide access to social and financial services, as well as national and export markets, and can empower small-scale rural producers.

The legal status of the women’s micro-credit fund: Rural women’s micro-credit funds have been excluded from the banking system. Start-up funds not only should create an environment for rural women to manage financially their businesses, but also to engage in business activities that are traditionally male-dominated. The question is whether microfinance credit funds can enable women to take action to prevent financial constraints in rural areas.

Remove systemic barriers to improve women’s access to funds: The business regulatory process should be simple, cost-effective, and consolidated. It is not clear to see how rural women are taking advantage of microcredit funds. Rules and regulations governing the distribution of financial facilities by micro-credit funds can provide opportunities or threats for rural women.

Market access: The ability of rural women to negotiate fair prices for products and services depends on their access to new markets. Rural areas, due to a lack of access to new markets, remain trapped in a subsistence economy in which rural women’s production systems cannot function effectively and efficiently.

### 3.4 MDT strategies

The participant cited 10 sub-themes as market-driven strategies and the most prevalent of them were “participatory training to rural women (participatory method)” and “organizing rural women.”

One interviewee expressed that the right capacity and support mechanisms must be available locally to establish competitive markets in rural areas. To succeed in local and national markets rural women training programs should enclose competitive market-oriented strategies to manage the relevant demand and risk.

One participant said: when a rural woman going to start a business, she will face systematic barriers. So, rules and regulations should be reformed to provide access to those rural women who have been excluded from opportunities in rural.

### 3.5 Consequences in MDT

According to the analysis, key consequences associated with the MDT program for rural women were found to be reversing the migration process, empowering rural women, increasing customer choice, and improving the quality of products. The followings are statements about market-driven training consequences:

The market plays a basic role in rural household welfare. The livelihoods of rural households depend directly on their involvement in markets, either as producers or workers. The market training program can have considerable effects on the diversification strategies of rural households’ income when disasters occur. They must deal with homelessness, poverty, and violence, which contribute to their impoverishment as well as their children. The content of various market-driven training programs must cover areas such as financial issues. If the market-driven training program is focused on helping customer satisfaction, this would grow the sales of their businesses and eventually improve their livelihood.

## 4 Discussion

To develop a model, grounded theory was used to explore the MDT for rural women in the Kurdistan province, Iran. According to Kim et al. (2009), GT is a middle-range theory to uncover structures of meaning and to increase transparency by the individual to explain the purposes and process (Kim et al., 2009; Wagner et al., 2010). Recognizing the popularity of GT in social sciences (Pulla, 2016; Milani and Hashemi, 2020; Sharma et al., 2020), it can be used as a paradigm to define opportunities and threats facing rural businesses (Milani and Hashemi, 2020).

The current study tried to manifest the strategy extension agents use when they want to implement market training programs for rural women. Based on the results of the study, seven categories were identified that describe MDT in the Kurdistan rural area. The results clarified the roles that rural women can play in MDT programs through casual, contextual, and intervening conditions. The core category that emerged from data analysis, in line with previous research, confirms the importance of designing and implementing market training for rural areas. The study showed that access to the market, funds, and monitoring of activities as contextual factors, could play a significant role in the market-driven training program for rural women. Despite various training programs in the rural area of Kurdistan, MDT faced a major challenge in linking farmers to input and output markets. In this regard, Dutta and Banerjee (2018) and Ganle et al. (2015) suggest that access to capital has a significant role in entrepreneurial activities.

On the other hand, culture as the most intervening condition shows the importance of cultural factors in developing agricultural extension programs and designing MDT programs, to provide better access to the market. According to Seuneke and Bock (2015), culture defines the level of participation of rural women. The study by Chan and Liu (2008) on Women and rural livelihood training in the Integrated Agriculture Training Program (IATP) in Papua New Guinea reported considerable success in implementing appropriate training for women, meeting the needs of rural women, and making positive impacts on women’s livelihoods, but its success was hindered by not responding to gender concerns.

The study showed that the casual conditions include two categories in the qualitative study. These conditions are supported by existing literature in the context of continuous education, financial support, and contract farming (Alavion and Taghdisi, 2020; Savari et al., 2020). Additionally, strategies should cover gender-sensitive policies, participatory training, and organizing rural women. To make these strategies successful in empowering rural women, it is important to attract and enhance the participation of trainees (Coles and Mitchell, 2010). As Oduol et al. (2017) suggested that to improve the status of rural women in male-headed households, there is a need to institutionalize gender-sensitive policies in the management of producer groups. In a study by Kozica et al. (2015) on programs for rural women, the results show that barriers to participation in rural communities included lack of status, self-consciousness, and social segregation. Participants also reported that exploiting local support and maximizing public attention is fundamental to strengthening the participation of rural women in future programs.

MDT should enable the diversification of skills and transform the low-skilled labor forces into high-skilled ones to improve market competitiveness. The studies showed that there was a positive relationship between educational attainment and improved skills (Alavion and Taghdisi, 2020; Savari et al., 2020).

This is particularly apparent in reversing the migration process, empowering rural women, increasing customer choice, and improving the quality of products (Devaux et al., 2009; Hoa et al., 2018). Sell and Minot (2018) reported that there were individual and household characteristics associated with women’s empowerment. Age and education are associated with empowerment, but equality in providing training for males and females is more important than the average level of education. Economic empowerment of rural women is essential, not only for the well-being of individuals, families, and rural communities but also for overall economic productivity, given women’s large engagement in the agricultural small-scale business. For the economic benefits from training, research shows that at each additional level of educational attainment there are improved market outcomes for individuals (Alavion and Taghdisi, 2020; Savari et al., 2020). Based on the results of the studies by Micheels and Gow (2014); and Mirdamadi et al. (2016), market needs assessment, production needs, responsiveness to the customers, and participatory rural women need assessment have been considered as key determinates of market training program to rural women. So, agricultural extension agents must pay more attention to cultural factors and norms about Kurdish rural women in designing MDT programs.

Educational services have direct and indirect benefits for rural population who have been affected by Covid. This is no exception for market driven training which has the potential in offering educational services to the rural women. A study by Mueller et al. (2021) about the impact of Covid on rural population in America, shows that effects on rural populations have been severe, with significant negative impacts on unemployment, overall life satisfaction, mental health, and economic outlook. These impacts have been generally consistent across age, ethnicity, education, and sex.

## 5 Conclusion

Cultural norms in the Kurdistan Region significantly impact rural women’s participation and success in training programs. While traditional gender roles prioritize household responsibilities, limiting time and permission for participation, these programs offer an opportunity to empower women. Cultural expectations of female deference can lead to lower confidence and hesitation to participate actively. Additionally, restrictions on women’s mobility, particularly unsupervised travel, can hinder attendance at programs located far from villages.

However, these challenges can be addressed. Training programs can foster strong community networks among participating women and their families. By focusing on practical skills that directly address women’s immediate needs and income generation opportunities, programs can increase motivation and perceived value. The evolving social landscape of the Kurdistan Region, with growing educational opportunities and women’s rights movements, presents a window for positive change. Culturally sensitive program design that respects local customs while promoting gender equality can contribute to this progress.

Intensifying rural women’s access to new markets is a high priority in developing a framework that enables market-driven training to serve as a key driver for rural women’s economic growth. The research recognizes that there is a need for a more practical and inclusive approach toward market-oriented training. This can be achieved by integrating the strengths and application of existing legal capacities along with participation of experts in developing the training programs. This would facilitate the process of the empowerment of rural women.

It is important to engage rural women in both on-farm and off-farm activities to ensure their families can access food and diversify income sources. In other words, they often suffer more than men because of their limited access to resources and income. Policies should address these gender inequalities, as well as the special roles and needs of rural women. There is a need for a more gender-responsive policy environment that facilitates the legislative and policy reforms for enforcing better and effective access for startup businesses run by rural women to micro-credit funds. Access to services, such as basic energy, water infrastructure, and childcare services by the public sector would lead to more trust in the government. The regulations must facilitate assistance from the financial sector to reach more rural women with better interest rates, loan options, and financial literacy training (Zobeidi et al., 2021).

Building strong rural women’s organizations is important to strengthen the capacity of rural women entrepreneurs to advocate for and serve the needs of their members. These organizations can also support research about rural women’s market-driven training needs locally and regionally. Additionally, they can disseminate information on good practices in promoting sustainable businesses run by rural women.

Low educational levels, lack of training, and limited experience in business activities can limit the capacity of rural women to manage a business. Additionally, lack of information on job availability, limited access to technology and transportation, and lack of property rights are obstacles for rural women. There is a need for more advisory services for rural women to improve literacy, business skills, access to technology and markets, and increase their income to move out of poverty.

However, barriers such as lack of confidence by men in rural women’s abilities and reluctance of some rural women to step outside traditional roles require more emphasis on ensuring the local advisory market training programs to facilitate the process of capacity building and empowerment of rural women. Furthermore, the results showed that market-training programs should follow a multi-dimensional approach to achieve long-lasting results.

Sociologically, this study makes a significant contribution by focusing on a critical but under-researched population, rural women in developing economies. It sheds light on their unique challenges and opportunities, particularly regarding market-driven training programs. This focus is valuable because rural women are often overlooked in sociological research. Furthermore, the study moves beyond a narrow training-centric approach. It recognizes the need for a multi-dimensional strategy that tackles social, economic, and cultural barriers to empowerment. This aligns with contemporary sociological perspectives that view empowerment as a complex process requiring multifaceted interventions.

The findings from this study on cultural influences on training programs for rural women in Kurdistan can be valuable for improving policies and practices related to market-oriented training programs in identifying specific cultural barriers like limitations on time, mobility, and confidence that women face. This can inform policymakers and program designers to create culturally sensitive approaches. The research can showcase how strong community networks and addressing immediate needs can enhance participation. This can guide program design to foster supportive environments and address practical concerns.

The research can emphasize the importance of training that directly equips women with income-generating skills relevant to their local context. This can inform policymakers to prioritize practical skill development over generic training. The findings can highlight the need for strategies to boost women’s confidence and encourage active participation. This can lead to incorporating confidence-building workshops or mentorship programs within the training.

The research can provide evidence of the positive impact training programs can have on rural women’s livelihoods and potentially, the local economy. This can be used to advocate for increased government funding and support for such programs. The research can inform policies on how to involve community leaders, families, and local NGOs to garner support for training programs and encourage women’s participation.

Additionally, this research can have a broader impact in sharing the findings can inform the development of training programs in other parts of the world with similar cultural contexts. By showcasing successful strategies from this research (e.g., community-based learning, addressing immediate needs), it can contribute to a global knowledge base of best practices for market-oriented training programs for rural women.

## 6 Limitation

The practical consequences of this study are limited to market driven training for rural women in the Kurdistan province, so the model of training in this study is probably different in other provinces.

## 7 Further implication of the study

This study sheds light on how market-oriented training programs can empower and equip rural women for market participation. However, the generalizability of these findings to other regions might be limited due to cultural and contextual variations. Further research is needed to test the program’s validity in different settings. To broaden our understanding, future studies should explore the factors that hinder the development and effectiveness of market-driven training programs for rural women. This could include examining the impact of personal characteristics on training success and identifying critical determinants that ensure program sustainability.

## Data availability statement

The raw data supporting the conclusions of this article will be made available by the authors, without undue reservation.

## Ethics statement

The studies involving humans were approved by Maryam Omidi Najafabadi Science and Research Branch; Seyed mehdi Mirdamadi Science and Research Branch. The studies were conducted in accordance with the local legislation and institutional requirements. The participants provided their written informed consent to participate in this study.

## Author contributions

ZA: Formal analysis, Investigation, Methodology, Writing – original draft, Writing – review & editing. SH: Formal analysis, Investigation, Supervision, Validation, Writing – review & editing. FL: Supervision, Writing – review & editing. RM: Supervision, Writing – review & editing.
